# Decision times in orthographic processing: a cross-linguistic study

**DOI:** 10.1007/s00221-022-06542-0

**Published:** 2023-01-11

**Authors:** Marika Mauti, Chiara Valeria Marinelli, Richard J. O’Connor, Pierluigi Zoccolotti, Marialuisa Martelli

**Affiliations:** 1 Department of Psychology, Sapienza University of Rome, Rome, Italy; 2 Neuropsychology Unit, IRCCS Fondazione Santa Lucia, Rome, Italy; 3 Learning Science Hub, Department of Humanities, University of Foggia, 71122 Foggia, Italy; 4 Department of Psychology, University of Hull, Hull, UK; 5 Department of Systems Medicine, University of Rome Tor Vergata, Rome, Italy

**Keywords:** Cross-linguistic comparison, Lexical decision, Diffusion model, Face gender judgment

## Abstract

Reading comparisons across transparent and opaque orthographies indicate critical differences that may reveal the mechanisms involved in orthographic decoding across orthographies. Here, we address the role of criterion and speed of processing in accounting for performance differences across languages. We used binary tasks involving orthographic (words–pseudowords), and non-orthographic materials (female–male faces), and analyzed results based on Ratcliff’s Diffusion model. In the first study, 29 English and 28 Italian university students were given a lexical decision test. English observers made more errors than Italian observers while showing generally similar reaction times. In terms of the diffusion model, the two groups differed in the decision criterion: English observers used a lower criterion. There was no overall cross-linguistic difference in processing speed, but English observers showed lower values for words (and a smaller lexicality effect) than Italians. In the second study, participants were given a face gender judgment test. Female faces were identified slower than the male ones with no language group differences. In terms of the diffusion model, there was no difference between groups in drift rate and boundary separation. Overall, the new main finding concerns a difference in decision criterion limited to the orthographic task: English individuals showed a more lenient criterion in judging the lexicality of the items, a tendency that may explain why, despite lower accuracy, they were not slower. It is concluded that binary tasks (and the Diffusion model) can reveal cross-linguistic differences in orthographic processing which would otherwise be difficult to detect in standard single-word reading tasks.

**Supplementary Information:**

The online version contains supplementary material available at 10.1007/s00221-022-06542-0.

## Introduction

Word recognition in alphabetic orthographies is greatly influenced by orthographic depth. This is referred to either as transparency, regularity, or consistency of grapheme-phoneme correspondences. In regular orthographies, such as Italian or German, the correspondence is highly consistent and fully predictable based on ortho-tactic rules, while in irregular orthographies, such as French, Hebrew, or particularly English, it is not. Reading acquisition studies have shown that orthographic depth has an important influence on reading, particularly in the first stages of acquisition. In a large study examining 13 different European orthographies, reading accuracy at the end of grades 1 and 2 closely depended upon the degree of regularity of the orthography (Seymour et al. 2003).

Reading theories across languages that are concerned about the influence of orthographic depth on reading follow two general approaches whose main outcomes will be described in the next paragraphs. On the one hand, various studies examined the influence of psycholinguistic variables (such as length, body-neighborhood, and grain size) on reading in regular versus irregular orthographies. On the other hand, other studies have been based on componential analysis of the measures used to examine reading, in particular reaction times (RTs). These latter studies aim to identify the factors accounting for the global RTs and which of these would be differentially active in readers learning regular or irregular orthographies. In this vein, in the present study, our interest was in understanding whether differences in reading between languages with a highly irregular orthography, such as English, and a highly regular orthography, such as Italian, could be understood based on the application of the Diffusion model (Ratcliff 1978; Ratcliff and McKoon 2008), which provides a decomposition of the parameters influencing the overall response.

### Psycholinguistic studies

In a psycholinguistic perspective, Ellis and Hooper (2001) found that readers of Welsh (a transparent orthography) showed slower word reading RTs as a function of length as well as mispronunciation errors in the case of pseudoword stimuli. By contrast, English readers showed a very small length effect for words and word substitutions in the case of pseudowords. Ziegler et al. (2001) obtained similar results by presenting words and derived pseudowords to two groups of German, a transparent language, and English adults. German speakers exhibited a larger word length effect. English speakers, however, showed a larger body-neighborhood effect but no length effect: thus, they were faster in reading words sharing the same orthographic rhyme.

Developmental research investigating how these cross-linguistic differences emerge with age reports findings consistent with the above. Marinelli et al. (2016) observed that in Italian, a transparent language, reading relies initially on a small grain size, as indicated by the length effect, and progresses on to larger grain sizes with increasing age/experience. In contrast, English children rely on larger reading units from the beginning of reading acquisition, showing lower accuracy but faster RTs than Italian children, as well as a stronger lexicality effect and no length effect. However, considerable individual differences were present among English children such that approximately a fourth of them failed to develop parallel string processing and relied on sequential processing (with a strong length effect). Notably, large inter-individual variability in English was present also when English and Italian adult observers were compared using the rapid visual serial presentation (RVSP) paradigm (Marinelli et al. 2014). This finding indicates the importance of considering inter-individual variability and not only the overall group performance when looking at cross-linguistic differences. Overall, the reviewed studies appear broadly consistent with the idea that learning to read in a regular orthography (such as Welsh, German, and Italian) relies on smaller units (grapheme-phoneme), whereas readers learning an irregular orthography (such as English) early in their reading acquisition rely on larger units, such as body rhymes (Ziegler and Goswami 2005).

### Componential analysis of reading measures

A different line of research analyzed the components contributing to reading speed by modeling RTs, making use of the knowledge of the “laws” governing the RT measure (Wagenmakers and Brown 2007; Ratcliff 1978; Faust et al. 1999; Myerson et al. 2003). The RT distribution is generally skewed to the right, increases with task difficulty, and spreads with increasing means (Wagenmakers and Brown 2007). Furthermore, it is well known that RT means and standard deviations (SD) linearly increase with task difficulty. Models, such as the “rate-and-amount model” (RAM; Faust et al. 1999) or the “difference engine model” (DEM; Myerson et al. 2003), take these general laws into consideration to explain individual differences in performance on timed tasks. For example, according to DEM, the linear relation between SDs and task difficulty allows independent estimation of the decisional and non-decisional times in information processing (Myerson et al. 2003). The RAM proposes that individual performance largely depends on the multiplicative interaction of two basic factors: the amount of the information processing required by a given task (i.e., its difficulty or “amount”) and the individual general speed of responding (or “rate of processing”). Adopting a different perspective in interpreting RTs, the diffusion model developed by Ratcliff (1978; Ratcliff and McKoon 2008) aims to account for reaction times in two-alternative forced-choice tasks, such as lexical decision (word vs. pseudoword), proposing a decomposition of the different factors affecting overall performance (see below for a more in-depth description). This is the model that will be applied here to understand the reading differences between different orthographies.

It must be noted that RTs are considered as a reliable and informative way to understand mental processes if accuracy is high or optimal. However, it is also known that RTs for difficult conditions may be fast but associated with a high error rate (or vice versa). Such speed-accuracy trade-off effects would generally dampen the interpretation of findings (unless specific hypotheses are put forward; e.g., Cooper 1980). Across languages, results show that English readers are consistently less accurate than matched readers of regular orthographies, but the evidence is much more variable in the case of RTs. The cross-linguistic difference in accuracy data is particularly clear in developmental investigations (Welsh–English comparison; Ellis and Hooper 2001; Albanian–Greek–Hiragana-Kanji–English comparison; Ellis et al. 2004; German–English comparison; Ziegler et al. 2003; Dutch–English comparison; Patel et al. 2004; Italian–English comparison; Marinelli et al. 2016), while in adults, data on accuracy are sometimes close to ceiling, making cross-linguistic differences hard to detect (e.g., Ziegler et al. 2001; Marinelli et al. 2014). Considering RTs, English readers can be as fast as readers of transparent languages (Marinelli et al. 2014), faster at least in some conditions (Marinelli et al 2016) or ages (Ellis and Hooper 2001), and finally slower as well as less accurate (Ziegler et al. 2003; Patel et al. 2004). It is worth noting that in studies of functional reading where speed is measured as reading fluency across correct and incorrect responses, the percentage of errors can be quite high, particularly in languages such as English (e.g., Seymour et al. 2003), making these observations not informative as to the nature of processing speed as a function of orthographic consistency. Overall, English data appear at variance with the expected consistency between accuracy and speed measures, a pattern whose significance still awaits a coherent interpretation.

Mathematical models of RT measures have proven useful in interpreting cross-linguistic differences in reading. While most studies on reading consider the mean across participants, some authors (e.g., Yap et al. 2012) have investigated the possibility that these cross-linguistic differences may be in part driven by participants’ individual differences. Ellis and Hooper (2001) found the expected co-variation between condition means and SDs both in Welsh and English children. Marinelli et al. (2016) investigated reading acquisition in Italian and English children of different ages, applying RAM (Faust et al. 1999) and DEM (Myerson et al. 2003) models to interpret their results. They found that reading ability at different ages was the consequence of both the influence of specific factors—defined in terms of psycholinguistic variables, such as frequency and length—and of a global factor in information processing speed. However, there were marked differences in the way data of the two groups of children fitted the predictions of these models. Data from Italian children closely followed the models, i.e., there was a close relationship between means and SDs that enabled effective predictions based on a single, global factor. By contrast, data from English children did not clearly fit either model. In fact, compared to the Italian group, they were generally faster but more variable, showing a modest influence of task difficulty. Marinelli et al. (2014) compared the reading performance of English and Italian adults using the rapid visual serial paradigm (RSVP) and found that English participants read with a similar rate than Italian participants, but they showed a much greater inter-subject variability with very fast readers on the one hand and very slow readers on the other. In the same study, measures of vocal RT for reading were analyzed based on the ex-Gaussian distribution. Data showed a difference across groups in the size and variability of the exponential parameter (tau) and in the variance (sigma), but not in the mean (mu). These results were in keeping with the idea that the differences between the two language groups are dependent on the inter-subject variability in the distribution and not—or not only—on mean performances.

### Aims of the present study

Overall, cross-linguistic comparisons among orthographies of different depth offer several consistent as well as discrepant indications both in speed as well as accuracy measures, with English readers being generally less accurate but highly variable in terms of speed (e.g., Marinelli et al. 2020, 2016, 2014, 2016; Ziegler et al. 2003; Patel et al. 2004; Ellis and Hooper 2001). How can the general pattern of results be interpreted with reference to current models of RT performance? The quoted RT models (i.e., RAM and DEM) focus on task and individual parameters but do not consider other intervening variables on decision-making, namely the response criterion. Stemming from the classical psychophysical literature, Ratcliff (1978) has proposed and updated (e.g., Ratcliff and McKoon 2008) a diffusion model of RTs for fast two-alternative forced-choice decisions, which considers a starting point toward one of the two response criteria or boundaries (z), a criterion bias (boundary separation, i.e., the amount of evidence needed until a decision threshold is reached) along with a sensitivity parameter (drift, i.e., the rate with which the decision is made), as well as a non-decision component (Ter). This represents an important integration over the other quoted models (RAM, DEM) of individual performance in RTs. At the same time, it may be observed that the diffusion model has been devised for use with two-alternative forced-choice tasks (such as the lexical decision task), but it cannot be applied to other measures such as vocal reading RTs. Thus, one may anticipate that a set of paradigms and models may be necessary to fully account for the cross-linguistic differences among orthographies (a point that will be further developed in the general discussion). Here, we fit our experimental data using the EZ2 model (Grasman et al. 2009). EZ2 is an upgrade of the EZ method (Wagenmakers et al. 2007) that enables the application of the simplified diffusion model to binary choices where one response alternative is generally preferred over the other (such as the “YES” responses in lexical decision tasks), so it seemed particularly appropriate for modeling the present data.

The reviewed evidence indicates that the components that determine different RTs across different linguistic groups are not clear yet, and the described instability of group RT differences may indicate that multiple factors other than individual sensitivity may contribute to the observed variability. Evidence indicates that English subjects are prone to a more lexical type of processing based on larger reading units (Ellis and Hooper 2001; Marinelli et al. 2016; Ziegler et al. 2003). We suggest that this effect may be associated with a tendency in setting lower standards to reach a decision on the lexicality of an orthographic string. By contrast, readers of a regular orthography may rely on a more balanced lexical-and-sub-lexical mode of strategy, and their mode of processing may be more tightly associated to the characteristics of stimulus quality. To evaluate this hypothesis, we conducted a study to estimate the cross-linguistic differences in terms of drift rate and boundary separation (or response criterion) parameters, using the Diffusion model developed by (Ratcliff 1978; Ratcliff and McKoon 2008). In the first experiment, we compared the performance of an English and an Italian group of college students in a lexical decision task. According to the diffusion model, lexical decisions are made after the sufficient accumulation of noisy information about the letter string toward one of two choices or boundaries, in this case associated with a word or a non-word response (Ratcliff and McKoon 2008). In addition, according to the model, the criterion, or boundary separation, is independent from the rate of processing and set idiosyncratically to the task. Thus, based on the psycholinguistic studies discussed above, we predict that English readers, relying on larger reading units, enabling a parallel access to the lexicon, would use a more lenient criterion (lower boundary separations); on the contrary, Italian readers, relying on smaller reading units and on a more balanced lexical-and-sub-lexical mode of processing, would be more stimulus-bound and show a more conservative criterion (higher boundary separations). In a second control experiment, we examined the performance of a group of English and Italian young adults in a face gender discrimination task. We expect cross-linguistic differences to be selective for orthographic depth and as a result we predict the two groups not to differ in this control task.

## Experiment 1: lexical decision task

In this first study, we compared English and Italian young adults in a lexical decision test. In particular, we examined if cross-linguistic differences may be decomposed into different diffusion parameters: we wanted to evaluate whether English and Italian readers would differ in terms of criterion, drift or both. We also examined the role of word frequency. Previous studies have shown that lexical effects may be amplified in an opaque orthography (e.g., Marinelli et al. 2016). Accordingly, we examined whether English participants would show larger frequency effects than Italian participants.

## Methods

### Participants

Italian readers were 30 university students recruited from the student population of Sapienza University of Rome, while English participants were 29 students recruited from the student population of the University of Hull. Considering the diffusion model requirements, we eliminated from the analyses data obtained from two Italian participants who did not make any mistake in one of the experimental conditions (medium- and low-frequency words). The final sample of participants comprised 28 Italian and 29 English university students. The Italian group was composed of 14 females and had the mean age of 22.5 years, SD = 6.5 and 14 years of education; the English group was composed of 13 females and had the mean age of 22.3 years, SD = 4.29 and 14 years of education (all Fs were not significant). Participants had no records of learning disabilities or cognitive deficits.

### Materials

The stimuli used were 290 nouns and 290 pseudowords, with a length ranging from 6 to 7 letters in both languages. We wanted to assess the lexicality effect (words–pseudowords) and the frequency effect (medium- and low-frequency words) on reading RTs. As for Italian list, words were extracted from the Varless database (Burani et al. 2000; https://www.istc.cnr.it/grouppage/varless); for the English list, words were selected from the MRC Psycholinguistic Database2.0 **(**Wilson 1988; https://websites.psychology.uwa.edu.au/school/MRCDatabase/uwa_mrc.htm). Only Italian words with regular stress (i.e., on the penultimate syllable) and English words with regular correspondences (no letter-sound correspondence with a frequency of less than 5% according to Hanna et al. 1966) and regular plurals were included in the word sets. The sets in the two languages were balanced for ortho-syllabic difficulties (presence of double consonants, clusters of consonants and contextual rules), articulation point of the first phoneme, length (number of letters) and word frequency, but not for the neighborhood size, or N-size (Coltheart et al. 1977). The Italian N-size was calculated according to the Colfis database (Bertinetto et al. 2005) through the online program that can be found on https://lilia.dpss.psy.unipd.it/vicini/vicini2.php; the English one was calculated according to the Celex lexical database (Baayen et al. 1995) through the online program developed by Medler and Binder 2005; see http://www.neuro.mcw.edu/mcword/. In fact, N-size was higher in the Italian sets for both words (mean = 1.6) and pseudowords (mean = 0.5) than in English language (for words: mean = 1.3, *t* (289) = 2.26, *p* < 0.05; for pseudowords mean = 0.3, *t* (289) = 2.39, *p* < 0.05). However, it should also be considered that Italian has many inflectional forms (plural, masculine/feminine) that represent orthographic neighbors while these are fewer in English and do not necessarily represent neighbors (e.g., plurals almost always involve a change in the number of letters). Cross-linguistic differences were no longer significant if neighbors derived by inflectional morphology were excluded from the count of Italian neighbors.

Since to analyze data with the Diffusion model it is important that there is a substantial number of errors, we only used medium- and low-frequency words. We carried out both analyses on the full set of words (necessary to calculate reliable diffusion parameters) and on words separated in terms of word frequency. Due to difference in the numerosity of English and Italian database, frequencies values were reported to 1,000,000 occurrences in both languages, to have comparable values across languages. In the case of English, the low-frequency English set included words with a frequency lower than 1 (Mean = 0.64, SD = 0.42) (CELEX lexical database), while the medium-frequency set included words frequencies ranging from 9 to 111 (Mean = 39.27, SD = 37.76). The two sets did not differ for length (in both cases mean = 6.6, SD = 0.5, *F* < 1), N-size (for low-frequency words: mean = 0.7, SD = 0.9; for medium-frequency words: mean = 0.7, SD = 0.11; *F* < 1) and number of phonemes (in both cases Mean = 5.19, SD = 0.9, *F* < 1). In the case of Italian, the low-frequency set was made of words with a frequency lower than 1.9 (mean = 0.64, SD = 0.42), while the medium-frequency set included words frequencies ranging from 9 to 105 (mean = 35.86, SD = 36.65) (CoLFIS database). The two sets did not differ for length (in both cases mean = 6.6, SD = 0.5, *p* < 1), N-size (for low-frequency words: mean = 1.0, SD = 0.7; for medium-frequency words: mean = 0.9, SD = 0.8; *p* < 1) and number of words containing a stress on the antepenultimate syllable (*X*^2^ < 1).

Pseudowords in both languages were constructed by changing the vowels of each word in the word stimuli sets. The pseudoword stimuli therefore had the same syllabic structure and ortho-syllabic difficulties (i.e., presence of double consonants, cluster of consonants, diphthongs, and hiatuses, etc.) as the word stimuli, from which they were derived. The two sets of pseudowords (Italian and English) did not differ according to N-size, number of phonemes and number of letters (all *F*s < 1).

### Procedure

Participants were tested in a dimly lit room using a portable computer. They were seated 60 cm away from a computer screen. We wrote our experiments in Matlab, using the Psychophysics Toolbox extensions (Brainard 1997; Pelli 1997; Kleiner et al. 2007). Each trial began with a fixation point that remained on the screen for 200 ms. Subsequently, a word or a pseudoword appeared at the center of the screen. The stimulus remained on the screen until the participant pressed a keyboard key for the response. The subject was asked to decide as soon as possible the item lexicality by pressing the *c* key for words and the *n* key for pseudowords. 289 words and 289 pseudowords were presented in a fully randomized sequence within two blocks separated by a break. A set of 10 trials was administered prior to the experiment to familiarize participants with the task.

Reaction times (RTs) were recorded. RTs corresponding to errors and deviating from the individual subject mean by ± 5 standard deviations were excluded from the analyses.

### Data analysis

We first carried out a linear mixed effect model on raw RTs to correct responses with lexicality (words, pseudowords) and language group (English, Italian) as fixed effects and stimuli and participants as random effects. A logistic mixed effect model was performed on reading errors, with lexicality (words, pseudowords) and language group (English, Italian) as fixed effects and stimuli and participants as random effects. Analyses were replicated also with N-size as covariate. See Appendix 1 for more details.

Then, we applied the Diffusion model to the data to obtain estimates of the parameters (drift rate, boundary separation and starting position) for each participant. Here, we fit the data using the EZ2 method (Grasman et al. 2009; https://rdrr.io/rforge/EZ2/man/EZ2.cmrt.html). EZ2 is an upgrade of the EZ method (Wagenmakers et al. 2007) that enables the application of the simplified Diffusion model to choices which are unbalanced, i.e., subjects show a bias toward one response alternative (usually a “YES” response in lexical decision tasks). The diffusion model explains the cognitive processes that are involved in fast, simple two-choice tasks. It starts from the assumption that decisions are made through a noisy process that accumulates information from a starting point (*z*) toward one of the two alternatives. The quality of the information extracted from the stimulus determines the rate with which the decision is made (*drift rate, v*). The criterion or boundary separation indicates the amount of evidence needed until a decision threshold is reached: wider decision boundaries lead to slower and more accurate decisions, while narrower boundaries lead to faster but more error-prone decisions. The drift rate parameter was allowed to vary between words and pseudowords. The boundary separation parameter and the starting point were measured across the two alternatives. Individual means and variances of RTs to correct responses for the two alternatives as well as mean accuracy were the values used in the model.

In the case of a lexical decision task, the conditions’ choice (words–pseudowords) is not independent and is therefore typically interleaved in the experiments. This only partially controls the decision bias toward one of the two alternatives. EZ2 handles each experimental condition separately. It assumes that the decision processes associated with the two conditions (i.e., words and pseudowords) share the starting value *z* and the boundary separation a, with different drift rates. Note that the EZ2 model does not take into account the inter-trial variability for accurate and incorrect responses. Other methods, such as DMAT (Vandekerckhove and Tuerlinckx 2008) and fast-dm (Voss and Voss 2007), use more information from the RT distributions to draw conclusions about the decision processes (comparisons between fast-dm, DMAT, and EZ can be found in van Ravenzwaaij and Oberauerb 2009).

The observed responses are based on a moderate number of trials. Thus, we adopted two procedures to stabilize the parameters estimate. First as suggested by Voss et al. (2013), we looked at the individual time distributions and excluded the outliers. Second, in the individual fit, we kept the sensory motor component parameter constant; fixing this parameter makes the model more parsimonious and enhances the stability of the estimation procedure. Additionally, in above threshold, easy interleaved tasks as adopted here, it is unlikely that observers would be inclined to change the accumulation process or the criterion threshold during the run, resulting in more stable estimates. We let the drift rate, the boundary separation, and the starting choice position free to vary. Third, to obtain reliable estimates, we applied the model individually to the full set of words and pseudowords and performed separate ANOVAs on the estimated parameters. As indicated by Grasman et al. (2009), the drift rate is assumed to be normally distributed around the mean and a starting position uniformly distributed around the estimated z value. This motivated the analysis choice (e.g., Horn et al. 2011; Boywitt and Rummel 2012; Ratcliff 2013). A power analysis was performed through G power (Faul et al. 2009) to check for the adequacy of the sample size, which revealed that no correction of the F cut-off was necessary.

Since we were also interested in examining the possible role of word frequency in modulating cross-linguistic differences, we carried out a further linear mixed effect model on raw RTs on correct responses comparing RTs to medium-frequency and low-frequency words. As the effect of frequency is clear in the case of words but not in the case of pseudowords, where frequency simply refers to the word from which the pseudoword is derived, we carried out the analysis limited to word items with frequency (medium, low) and language group (English, Italian) as fixed effects and stimuli and participants as random effects. A logistic mixed effect model was performed on accuracy score, with frequency (medium, low) and language group (English, Italian) as fixed effects and stimuli and participants as random effects. Analyses were replicated also with N-size as a covariate. See Appendix 1 for more details.

## Results

Table 1 reports means and standard deviations of the RTs for correct responses and errors in the lexical decision task for each group of participants. An inspection of the RT means indicates that, as expected, in both languages, responses to words were faster than responses to pseudowords. Observing the percentages of errors, it is evident that both groups made more errors identifying a word as a pseudoword. Furthermore, English participants tended to be faster but also made more errors than Italian participants.

**Table 1:** Means and standard deviations (SD) of RTs and percentages of errors in lexical decision task

	Italian participants		English participants	
	Mean	SD	Mean	SD
Word RTs (ms)	867	152	846	137
Pseudoword RTs (ms)	997	242	930	157
Word errors (%)	7.8	4.6	12.8	5.1
Pseudoword errors (%)	4.5	2.6	7.1	5.4

Data are separately presented for words and pseudowords and for the two groups of participants

**Fig. 1:**
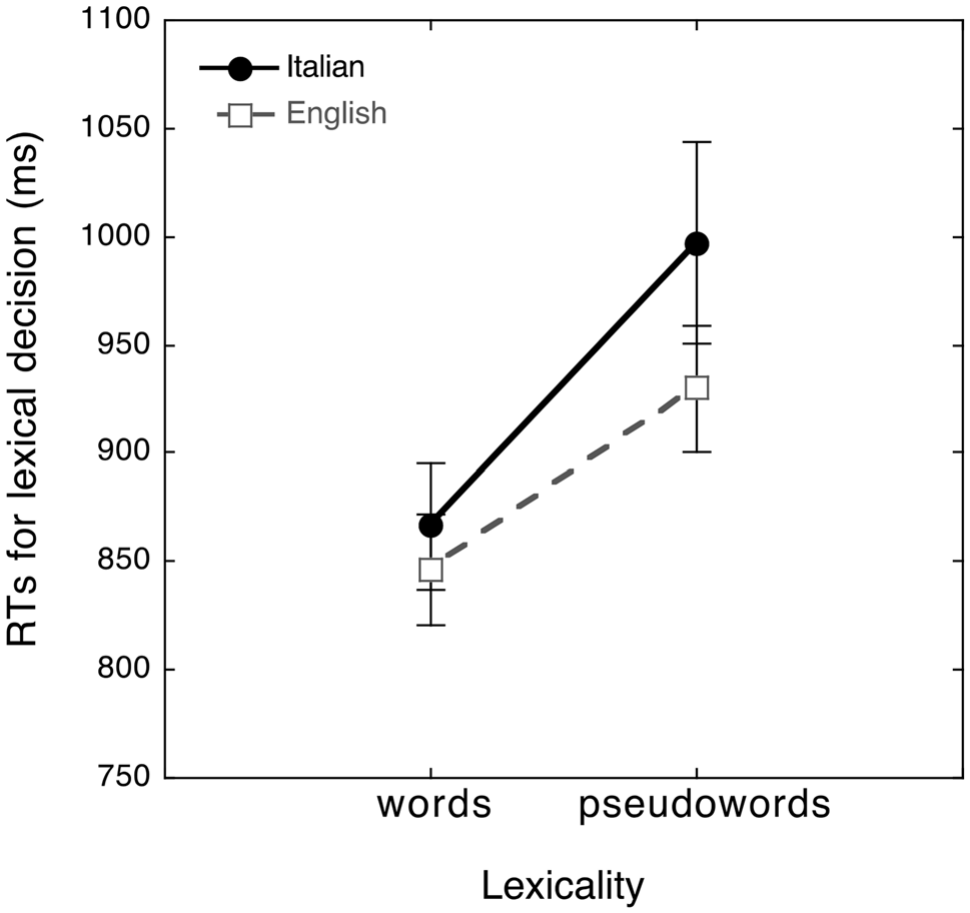
Mean and standard errors of raw RTs for Italian (solid circles) and English (empty squares) participants

The linear mixed effect model performed on raw RTs showed the significance of the lexicality effect (*t*_(1088)_ = 11.05, *p* < 0.0001), with faster RTs for words (emmeans = 870 ms, SE = 23.3) than pseudowords (emmeans = 968 ms, SE = 23.2) and no significant difference between language groups (emmeans = 938 and 900 ms for Italian and English participants respectively, *t* < 1, n.s). The lexicality by language group interaction was significant (*t*_(1102)_ = 3.36, *p* < 0.001; see Fig. 1). An inspection of the relevant means indicates that the lexicality effect was significant in both groups (at least *p* < 0.0001), but with a greater difference between words and pseudowords in the Italian (emtrend = 125 ms, *z* = 11.05, *p* < 0.0001) than in the English sample (emtrend = 71 ms, *z* = 6.31, *p* < 0.0001). The random effects of participants (*Z* = 5.21, *p* < 0.0001) and stimulus (*Z* = 18.46, *p* < 0.0001) were both significant. When the analysis was carried out again controlling for N-size, the N-size covariate was not significant (t about 1, n.s.), and results were replicated: the lexicality effect (*t*_(1087)_ = − 9.77, *p* < 0.0001) was significant; the language effect was not significant (*t* about 1, n.s.) but interacted with lexicality *t*_(1097)_ = 3.19, *p* < 0.001). The logistic mixed effect model performed on errors showed a main effect of lexicality (*z* = − 2.84, *p* < 0.01), indicating more probability to make an error misidentifying pseudowords (emmean = 3.93%, SE = 0.12) than words (emmean = 3.44%, SE = 0.12). The effect of language group was significant (*z* = 1.93, *p* < 0.05): English observers have a higher probability to make errors (emmean = 3.91, SE = 0.14) than Italian ones (emmean = 3.46, SE = 0.14). The lexicality by language group interaction was not significant (*z* < 1, n.s). The random effect of participants (*S* = 3048, *p* < 0.0001) was significant. When the analysis was carried out again controlling for N-size, the N-size covariate was not significant (*t* < 1, n.s.), and results were replicated: the lexicality effect (*z* = 2.47, *p* < 0.01 and the language effect (*z* = 1.95, *p* < 0.05) were significant, but not their interaction (*z* < 1, n.s.).

### Diffusion model

We examined the effect of language on the three diffusion parameters: drift rate (speed of processing); *z* value (bias toward one of the two alternatives); and boundary separation (amount of information set by the participant to accept one of the two alternatives in the decision process).

**Fig. 2:**
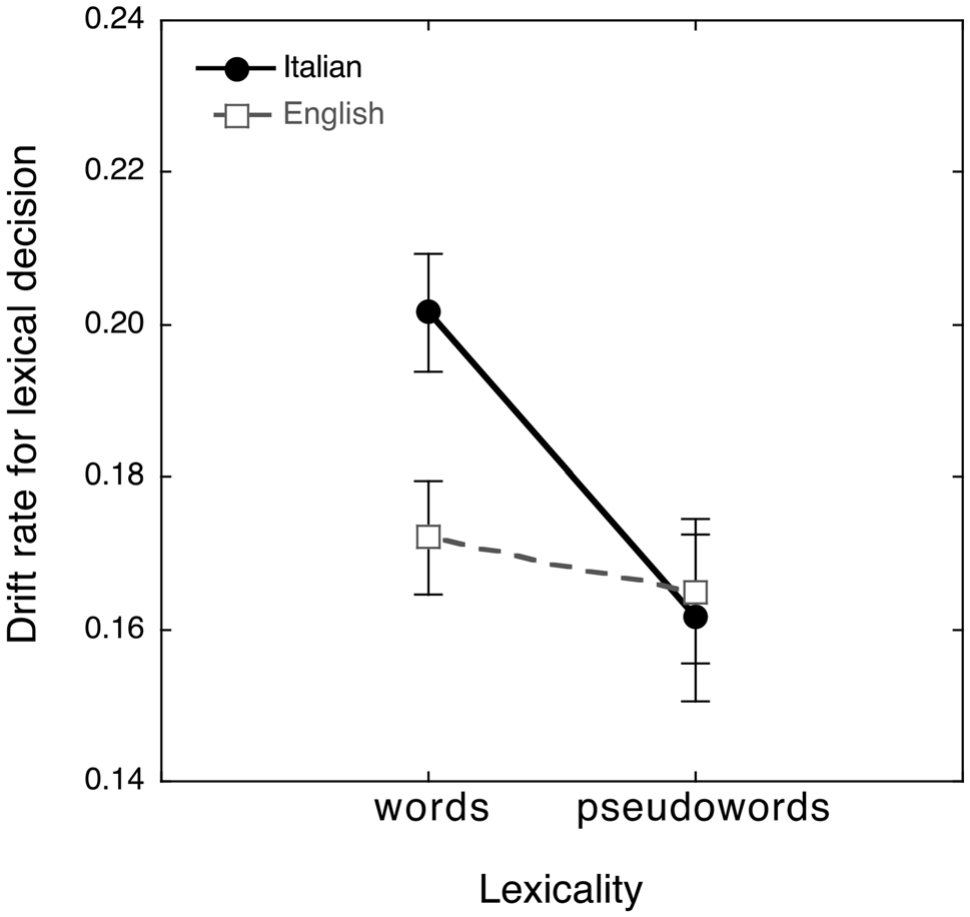
Mean and standard errors of drift rate for Italian (solid circles) and English (empty squares) participants

The ANOVA on drift rates showed a main effect of lexicality (*F*_(1,55)_ = 12.01 *p* = 0.001), with higher values for words (mean = 0.19, SD = 0.04) than for pseudowords (mean = 0.16, SD = 0.05). The effect of language group was not significant (*F*_(1,55)_ = 1.53, n.s.): drift rate averaged 0.17 and 0.18 for the English and the Italian group, respectively. The lexicality by language group interaction was significant (*F*_(2,55)_ = 5.86, *p* < 0.05), indicating that the advantage in responding to words over pseudowords was significant in Italian observers (diff. = 0.04; *p* < 0.01) but small and not significant in English observers (diff. = 0.01, n.s.; see Fig. 2). Groups differed in the case of words, with higher drift rates for Italian than English participants (difference = 0.03; *p* < 0.0001), but not in the case of pseudowords (difference = 0.00; n.s.).

The analysis on z values indicated no significant difference between the two groups (*F*_(1,55)_ = 2.27, n.s.): z values averaged 0.07 (SD = 0.018) for the Italian group and 0.06 (SD = 0.014) for the English one.

**Fig. 3:**
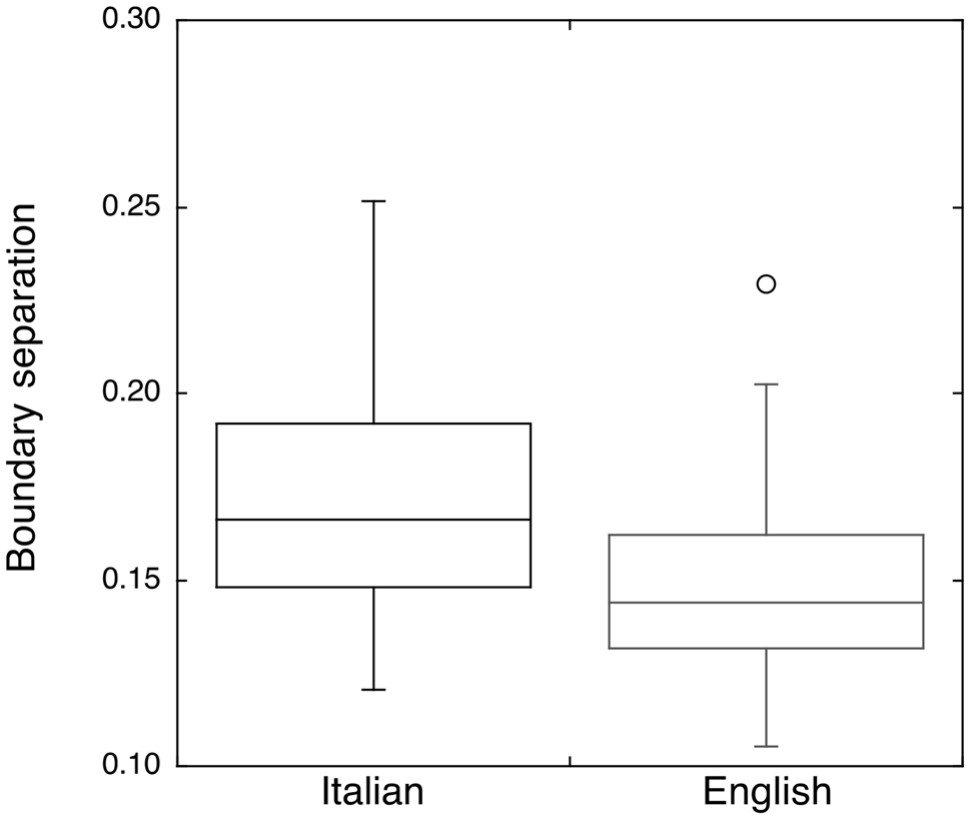
Boundary separation values for Italian and English participants. The boxplot represents the mean, variance and outliers. The horizontal lines represent the means for the two groups

In the analysis on boundary separation values, the language group effect was significant (*F*_(1,55)_ = 9.95; *p* < 0.01), indicating lower boundaries for English (0.15, SD = 0.02) than for Italian readers (0.17, SD = 0.03). Figure 3 shows both the individual and group mean values for boundary separation in the two language groups.

### The effect of frequency

Table 2 reports the means (and standard deviations) of RTs and the mean percentages (and standard deviations) of errors taking into account the effect of frequency. The table presents values for lexical decisions on words for each group of participants separately for medium-frequency (MF) and low-frequency (LF) words. In both languages, participants made faster decisions for medium-frequency than low-frequency words.

**Table 2:** Means and standard deviations (SD) of RTs and percentages of errors in lexical decisions for medium- and low-frequency words

	Italian participants		English participants	
	Mean	SD	Mean	SD
Medium-frequency (MF) words: RTs (ms)	819	149	776	116
Low-frequency (LF) words: RTs (ms)	920	159	936	168
Medium-frequency (MF) words: errors (%)	2.3	2.4	2.5	2.3
Low-frequency (LF) words: errors (%)	13.2	7.4	23.0	8.5

Data are separately presented for the two groups of participants

Data on errors indicate that both language groups made many more errors for low-frequency words than medium-frequency words. This was particularly noticeable among English participants; indeed, they showed a large discrepancy in the percentages of errors made between medium-frequency and low-frequency words**.**

**Fig. 4:**
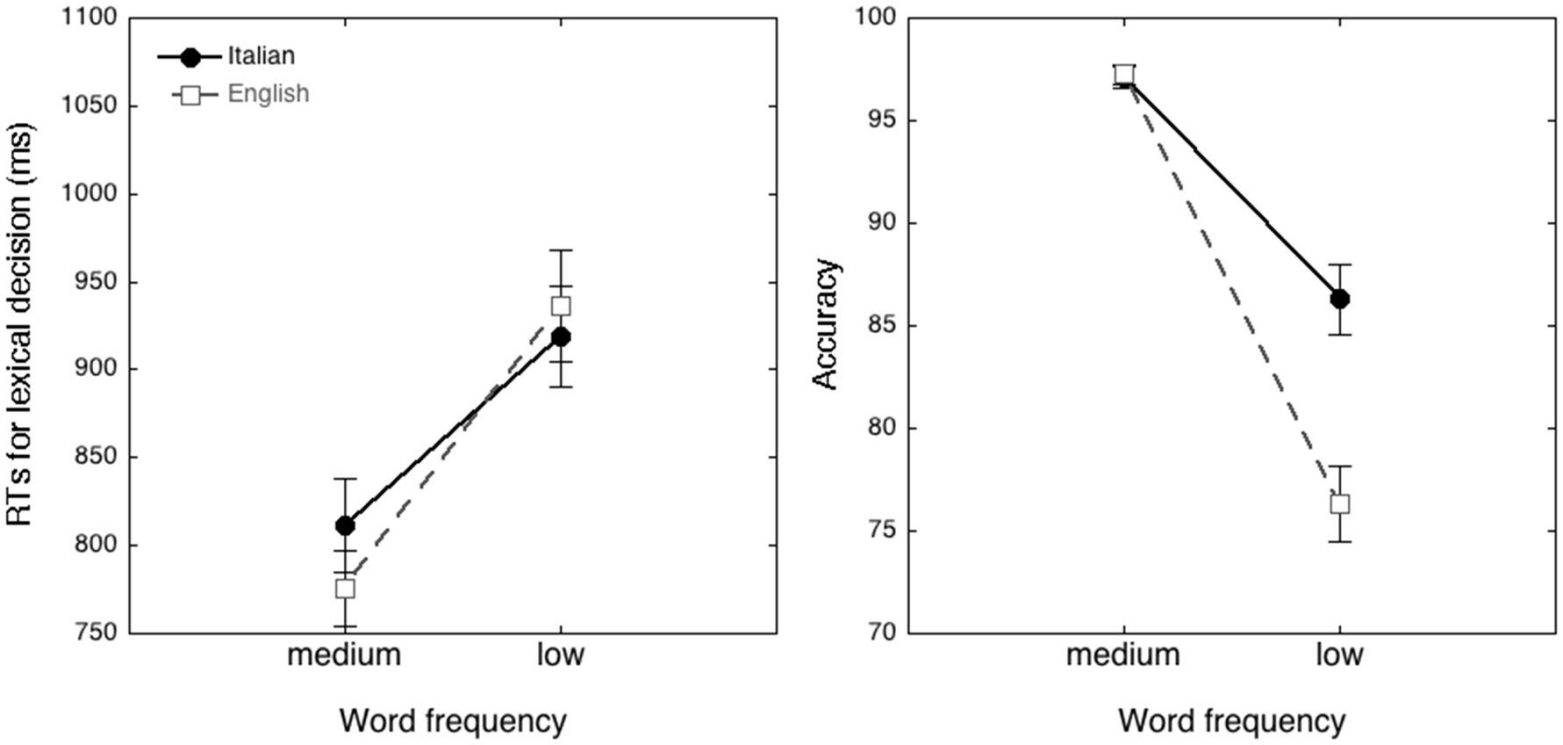
RTs (left) and accuracy (right) and error bars for words as a function of word frequency (medium–low) for Italian (solid circles) and English readers (empty squares)

The linear mixed effect model on RTs showed a main effect of frequency (*t*_(1120)_ = 4.91, *p* < 0.0001), with faster RTs for medium-frequency words (emmean = 881 ms, SE = 23.3) than low-frequency words (emmean = 959 ms, SE = 23.3). There was no significant difference between language groups (*t* < 1, n.s.): emmean for the English group RTs is 902 ms (S.E. = 32.2) while for the Italian one, 938 ms (SE = 32.7). The frequency by language group interaction was significant (*t*_(1135)_ = 2.48, *p* < 0.01; see Fig. 4a). An inspection of the means indicated that the frequency effect was larger in the English (emtrend = 98.6 ms, S.E. = 11.7, *z* = 8.41, *p* < 0.0001) than in the Italian (emtrend = 57.5 ms, SE = 11.7, *z* = 4.91, *p* < 0.0001) sample. The random effects of participants (*z* = 5.16, *p* < 0.0001) and stimulus (*z* = 12.49, *p* < 0.0001) were both significant. When the analysis was carried out again controlling for N-size, the N-size covariate was significant (*t*_(1104)_ = 6.19, *p* < 0.001), but results were replicated: the frequency effect (*t*_(1112)_ = 4.77, *p* < 0.0001) was significant, the language effect was not significant (*t* < 1, n.s.) but interacted with frequency (*t*_(1126)_ = 2.66, *p* < 0.01).

The logistic mixed effect model on errors showed a main effect of frequency (*z* = 7.15, *p* < 0.0001): the probability to make an error increased from 2.98 (SE = 0.11) for medium-frequency words to 4.45 (SE = 0.13) for low-frequency ones. The effect of language group was significant (z = 2.84, p < 0.01), indicating a higher rate of errors in the English (emmean = 3.96, SE = 0.15) than in the Italian (emmean = 3.47, SE = 0.14) sample. The frequency by language group interaction approached significance (*z* = 1.68, *p* = 0.09; see Fig. 4b): English and Italian participants had a similar probability to be inaccurate in the case of medium-frequency words (emtrend = 0.22, SE = 0.24, *z* < 1, n.s.), while they differed for low-frequency words (emtrend = 0.63, SE = 0.22, *z* = 2.84, *p* < 0.05), with a higher probability to be inaccurate in the English group (15.1%) than in the Italian one (7.7%), and larger frequency effect among English participants (emtrend = 1.67, SE = 0.17, *z* = 9.83, *p* < 0.0001) than Italian ones (emtrend = 1.26, s.e. = 0.18, *z* = 7.15, *p* < 0.0001). The random effects of participants (*z* = 4.61, *p* < 0.0001) and stimulus (*z* = 11.76, *p* < 0.0001) were both significant. When the analysis was carried out again controlling for N-size, the N-size covariate approached significance (*z* = 1.80, *p* = 0.07). The frequency effect (*z* = 7.20, *p* < 0.0001) was significant, as well as the language effect (*z* = 3.07, *p* < 0.01), while the language by frequency interaction approached significance (*z* = 1.60, *p* = 0.10).

### Synthesis of findings

English participants made more errors than Italian participants particularly in the case of low-frequency words. By contrast, they showed generally similar RTs and, if anything, a slight though not significant tendency to be faster and not slower than Italian subjects. This asymmetry is consistent with what is reported in the literature briefly reviewed in the introduction. The pattern of data for accuracy and time measures calls for the need of an explanation of why the processing of English participants is not slower despite being considerably less accurate.

Apart from group differences, it should be observed that the experimental manipulations in Experiment 1 were successful in producing a sufficiently large number of errors in most observers, a pre-condition for the application of the diffusion model. The results from the Diffusion model indicated that the two groups of subjects were not different in terms of *z* values, as one might expect. They were also not globally different in terms of drift values, although they showed a different pattern for word and pseudoword decisions: English participants showed lower drift values for word (but not pseudoword) decisions than Italian observers as well as a smaller difference between words and pseudowords. Furthermore, the two groups were different in terms of boundary separation with the English participants having in general lower boundaries. This indicates that English participants set their decisional standards at a lower level, i.e., they showed less conservative criteria than Italian observers. This tendency may well explain why, despite their lower accuracy level, they were not slower. By setting lower standards to accept the stimulus as either a word or a pseudoword, English participants were able to reach a speed which is not in keeping with the capacity that would be predicted from their lower accuracy measures. This lower criterion may well be an indication of differences in strategic approach in word reading. The nature of the drift and criterion differences will be examined in greater detail in the General Discussion.

The analyses of data as a function of frequency indicated a greater frequency effect among English participants. In general, the frequency effect is a marker of reliance on the lexical reading route, or it reflects the availability of the representation within the individual’s orthographic lexicon. Thus, this finding is in keeping with the idea that reading in English plays a greater emphasis on lexical processing.

## Experiment 2: gender judgment test

In Experiment 2, we compared English and Italian young adults on a face gender judgment test. This experiment served as a control of Exp. 1. Using faces as target stimulus seemed appropriate as faces share with words several features (i.e., they are both recognized by parts: letters and facial features, respectively) although they lack orthographic information (Martelli et al 2005). The gender judgment task was chosen as a simple two-choice task that would fit well with the requirements of the Diffusion model. We anticipated that, if the differences in decision making between the two linguistic groups are specifically related to differences in orthographic depth between the two languages, there should be no differences in critical diffusion parameters in this non-orthographic task. In view of the results of Experiment 1, we examined whether the two groups were different in boundary values to establish whether the lower criteria observed in Experiment 1 are specific to an orthographic task.

## Methods

### Participants

We tested 25 Italians and 23 English-speaking participants recruited from the student population of Sapienza University of Rome and from the University of Hull, UK. Since the diffusion model assumes that decisions necessarily imply some error, two English and four Italian participants who did not make any error were excluded from further analyses. The final sample of participants comprised 21 Italian and 21 English University students. The two groups were matched for age and educational level. The English group was composed of 18 females and had a mean age of 21.95 years, SD = 9.29 and 14 years of education. The Italian group was composed of 12 females and had a mean age of 20.45 years, SD = 4.29 and 14 years of education (all Fs not significant).

### Materials

The stimuli used were 20 faces (10 male and 10 female) selected from the Radboud Faces Database (RaFD; https://rafd.socsci.ru.nl/RaFD2/RaFD?p=main; Langner et al. 2010). Ten female and ten male faces were selected from the neutral expression subset. Faces were presented on a black background and shown through a Gaussian window of luminance profile to eliminate information other than the facial features (i.e., the hair was filtered out). Face width measured 4 deg of visual angle.

### Procedure

Participants were tested in a dimly lit room using a portable computer. They were seated 60 cm from the computer screen. We wrote our experiments in Matlab, using the Psychophysics Toolbox extensions (Brainard 1997; Pelli 1997; Kleiner et al. 2007). Each trial began with a central fixation point that remained on the screen for 500 ms. Subsequently, a face appeared in the same position. In each trial, the stimulus was randomly sampled from the set. Each face appeared for a maximum of 23 times. The stimulus remained on the screen until the participant responded. Participants were asked to perform a gender discrimination as quickly as possible by pressing the N key if the stimulus was a male face or the V key if the stimulus was a female face. Five blocks of stimuli with 60 trials each for a total of 300 trials were presented. RTs were recorded. RTs corresponding to errors and RTs exceeding the individual mean ± 5 standard deviations were excluded from the analyses.

### Data analysis

Like Experiment 1, we first carried out a linear mixed effect model on raw RTs to correct responses and a logistic mixed effect model on accuracy scores. Because face stimuli were repeated across the experiment (while in experiment 1 each stimulus was presented only once), analyses were performed controlling for the effect of item repetition. Then, language (English, Italian), face gender (male, female), and order of stimulus repetition were entered in the analysis as fixed factors, while items and participants as random factors.

Then, we applied the diffusion model to the data to obtain estimates of the parameters (drift, boundary and starting position) for each participant. The drift rate parameter was allowed to vary between male and female faces. The boundary separation parameter and the starting point were measured for the binary decision between male and female faces. Separate ANOVAs were carried out to examine the language effect on drift, *z*, and boundary values. Due to the small number of participants, the cut-off for the significance of F was corrected according to results of power analysis (G power 3.1, Faul et al. 2009). According to this correction, an F lower than 2.49 was considered not significant.

## Results

Table 3 reports the mean and standard deviation of RTs and percentages of errors of face gender recognition for each group of participants.

**Table 3:** Means and standard deviations (SD) of RTs and percentages of errors in face gender recognition for each group of participants

	Italian participants		English participants	
	Mean	SD	Mean	SD
Male face: RTs (ms)	651	122	724	123
Female face: RTs (ms)	716	160	763	117
Male face: errors (%)	3.9	4.7	4.6	3.6
Female face: errors (%)	4.7	5.4	7.0	5.2

The mixed effect model on RTs showed that the main effect of face gender was significant (z = 4.62, p < 0.0001), indicating faster responses for male (emmean = 683 ms) than for female (emmean = 726 ms) faces. The effect of language group was not significant (*z* about 1, n.s.): emmean RTs were 678 ms for the Italian observers and 732 for the English observers. The effect of order was significant (*F*_(23,11117)_ = 3.44; *p* < 0.0001, range *z* = − 1.68 to + 5.63), indicating a progressive reduction of RTs for each presentation of the same face, as well as the order by gender interaction (*F*_(23,11124)_ = 1.88; *p* < 0.01, range *z* = − 5.43 to + 2.80), indicating a progressive reduction of the gender effect after each presentation of the same face. The face gender by language group interaction, as well as any other interaction with language, was not significant. The random effects were significant (items: *S* = 69.36 *p* < 0.0001; participants: *S* = 549.84, *p* < 0.0001).

The mixed effect model on errors showed that the main effect of face gender was not significant (*z* = 0.03, n.s.), indicating similar percentages of errors for male (4.23%) and female (4.16%) faces. The effect of language group was not significant (*z* = 0.002, n.s.): emmeans were 3.73% and 3.66% in Italian and English participants, respectively. Also, the order effect was not significant (*F*_(2,11907)_ = 1.17, n.s., range *z* = − 2.72 to + 2.58): there was no accuracy improvement as a function of the number of repetitions of the stimulus. The gender by language group interaction as well as any other interactions were not significant (zs < 1). The random effects were significant (item: *S* = 2.66 *p* < 0.01; participant: *S* = 3.54, *p* < 0.0001).

As for the diffusion parameters, the ANOVA on drift values showed that the main effect of the face gender was significant (*F*_(1,40)_ = 4.48, *p* < 0.05): the drift rate was 0.32 (SD = 0.19) for female faces and 0.28 (SD = 0.10) for male ones. The effect of language group was not significant (*F* = 1.39, n.s.): mean drift rate was 0.28 for the English and 0.33 for the Italian group. The gender by language group interaction was not significant (*F*_(2,40)_ = 2.23, n.s.): there was no difference in responding to a face gender over the other, so drift rate difference was about 0.07 for the Italian and 0.01 for the English group. The z value did not differ between the two language groups (*F*_(1,40)_ = 2.17, n.s.) with a mean of 0.10 (SD = 0.07) for the Italian group and one of 0.07 (SD = 0.04) for the English one. The boundary separation values were not significantly different between the two language groups (*F*_(1,40)_ = 2.37, n.s.): they were 0.28 (SD = 0.25) for the Italian subjects and 0.18 (SD = 0.16) for the English ones.

### Synthesis of findings

In general, the gender face task was effective in producing enough errors in most observers, a pre-condition for carrying out the analyses in terms of diffusion parameters. There was an unexpected difference in RTs between male and female faces, where female faces were identified slower than male ones. Since there is no gender difference in the subject sample predicting the effect, this may be due to the discriminability in the stimuli set.

Apart from this effect, there were no significant language group differences or interactions with the language group. The results from the diffusion model did not indicate any significant difference in drift rate and z value. In particular, the two groups were not different in terms of boundary separation, a finding in keeping with the idea that only in the case of orthographic tasks, English participants use a more lenient criterion to make a binary decision compared to other language groups with transparent orthographies.

## General discussion

The present results provide new and interesting information for understanding cross-linguistic differences in reading between individuals learning languages with different orthographic consistency. The main empirical findings concern a difference in the criterion, as estimated by boundary separation values according to the Diffusion model, and in the pattern of drift values as a function of word–pseudoword differences. As for the boundary values, English individuals showed a more lenient criterion in judging the lexicality of the items than did Italian individuals, at least in the case of adult expert readers examined in the present study. In terms of drift values, there was no overall cross-linguistic difference, but English observers showed lower drift values for word (but not pseudoword) decisions than Italian observers; they also showed a smaller difference in drift values between word and pseudoword decisions.

This pattern of findings, and in particular the presence of a lenient criterion, may help understanding why, even though they were less accurate, English observers were not slower in their lexical decision times (if anything slightly faster). In the introduction, we have briefly reviewed the evidence on the asymmetry between reading accuracy and speed in cross-linguistic comparisons. In brief, while evidence on accuracy consistently indicates a greater amount of reading errors in English observers compared to readers of transparent orthographies (Ellis and Hooper 2001; Ellis et al. 2004; Patel et al. 2004; Marinelli et al. 2016; Ziegler et al. 2003), results for reading speed are considerably more variable indicating no consistent trend (Marinelli et al. 2014, 2016; Ellis and Hooper 2001; Ziegler et al. 2003; Patel et al. 2004). Overall, it appears that the present results are broadly coherent with the findings in the literature, indicating that differences in criterion may contribute to understanding why, despite being less accurate, English observers are not slower readers. Furthermore, it is possible to speculate that differences in criterion may have contributed to the presence of a heterogeneous pattern of findings on reading speed, though the precise understanding of how differences in criterion may have interacted with the different experimental procedures used as well as with various individual parameters (including age, reading experience, and language) awaits further research.

The diffusion model is an effective general means to mathematically quantify performance in two-alternative forced-choice tasks (Bogacz et al 2006). However, it has been observed that the diffusion model (Ratcliff 1978; Ratcliff and McKoon 2008) does not make explicit representational assumptions on the nature of the underlying processes (for a discussion see De Moor et al 2005). Therefore, in understanding how differences in criterion may act in shaping subject’s responses it is also useful to refer to current psycholinguistic models of lexical decision. Indeed, it has been proposed that these two approaches can be seen as complementary (De Moor et al 2005). One influential psycholinguistic model is the multiple read-out model (MROM) by Grainger and Jacobs (1996). The original version of MROM proposed three processes as a base for speeded lexical decision responses. Two processes are intra-lexical: the activation level of the most activated word unit (M criterion), and the sum of the activation levels of all word units, an overall measure of ‘word-likeness’ (or cumulated evidence for a word—the Σ criterion). In this perspective, a positive YES response can be reached either because of activation of specific lexical entry or because of the sufficient activation of the Σ criterion, in keeping with the idea that the lexical decision does not necessarily rest upon the full identification of the target word (Grainger and Jacobs 1996). The MROM also posits a third process: time from the stimulus onset, to explain the production of non-word responses. The idea of a default deadline has been severely criticized by Wagenmakers et al. (2008) on several grounds, and particularly, on the observation that under specific conditions non-word responses can be faster, and not slower, than word responses, a finding difficult to reconcile with “deadline” models. In response of this, Dufau et al. (2012) have proposed a modification of the MROM such that the NO response is a constant value minus the evidence for a word (see also Grainger 2018 for an updated MROM + version of the model).

The idea that two different intra-lexical processes may act in performing a lexical decision may be critical to the understanding of cross-linguistic differences. The opacity of the English orthography (coupled with generally high levels of neighborhood density) may favor the adoption of a criterion based on general evidence, while the highly regular Italian orthography (coupled with the generally lower level of neighborhood density and with a very small number of inconsistent words that require the recovery of specific orthographic representations from the lexicon) may favor the adoption of a criterion based on evidence for a specific word. It may also be added that being stimulus-bound, the M criterion is generally believed to be fixed or invariant while the Σ criterion is more sensitive to stimulus characteristics (e.g., De Moor et al. 2005; Albonico et al. 2018), such as list context (e.g., Carreiras et al. 1997) and task demands (e.g., Grainger and Jacobs 1996). Therefore, the present interpretation is in keeping with the general idea of a greater strategic role in reading English as compared to languages with regular orthographies, such as Italian.

A formal equivalence between the diffusion model and the MROM is still to be determined (for a discussion see De Moor et al 2005). However, when seen in terms of the diffusion model, one may speculate that the cross-linguistic difference in reliance on general evidence versus evidence for a specific word would express itself in terms of different decisional criterion levels (for accepting the target as word or non-word), as well as in terms of a difference in sensitivity to responding to word stimuli (over non-word stimuli). As for the former, English individuals would adopt a less conservative criterion, yielding generally faster decision times associated with an increase of error responses (as it occurs with instructions to respond quickly at the expense of accuracy; Wagenmakers et al. 2008). By contrast, observers of languages with regular orthographies, such as Italian, may adopt a more conservative criterion, associated with slower RTs and fewer errors. As to the latter, it is proposed that Italian individuals rely more on word specific (than on general) lexical evidence; accordingly, this should favor the sensitivity of the decision for words (as specific word evidence is selectively used for this decision) and not that for pseudowords which is influenced by general evidence only (Grainger 2018). Conversely, for English participants, it is more parsimonious to rely more on general than on word specific evidence, due to the high number of words requiring lexical processing. For this reason, English individuals may show a less pronounced advantage for word decisions. The present experimental data are in keeping with this proposal. However, as this pattern was not anticipated, it seems important that it is confirmed by further independent research.

Critically, the presence of a cross-linguistic difference in response criterion was specific to an orthographic task and no difference was detected for a control task requiring the judgment of face gender. These data are in keeping with the idea that the adoption of a lenient criterion in English individuals is specific for tasks involving orthographic materials, presumably arising as an effect of learning to read this inconsistent orthography and does not represent a general individual characteristic. We used face stimuli as controls because, despite their surface differences, they share the sensitivity to the same parameters, i.e., familiarity and crowding, and are both recognized by parts (Martelli et al 2005). Still, the use of a gender discrimination task was largely chosen because its convenience in relationship to the adoption of the Diffusion model. Thus, it may be interesting in further research to extend the present results to other non-orthographic tasks. Furthermore, the relatively small sample tested with the control experiment represents a limitation of the present study and suggests caution in generalizing the results.

Providing a unitary interpretation of the cross-linguistic differences in reading skills (and probably also impairments) is made complex by the fact that different experimental paradigms are effective (or indeed necessary) to let group differences emerge between individuals learning an opaque (typically English) or more transparent orthographies (like Dutch, German, Italian, or Welsh). This idea draws on the proposal that no single paradigm is probably sufficient to account for all reading phenomena and “functional overlap” among different reading tasks may be instrumental to effective modeling (Grainger and Jacobs 1996). In the present study, we capitalized on the Diffusion model (Ratcliff 1978; Ratcliff and McKoon 2008) to obtain estimates of the criterion in responding. It has been noted that one limitation of models of RT performance (such as the RAM and the DEM) is that they are unable to capture this component of the response (for a discussion see Spieler 2001). At the same time, one should be aware that the diffusion model applies well to binary, two-choice tasks, such as the lexical decision task, while it cannot be directly applied to the classical reading task, where single words or pseudowords are presented, and vocal RTs are measured. Similar considerations may apply to the detection of individual differences in cross-linguistic comparisons. In previous work (Marinelli et al. 2014), we noted that large differences were present comparing English and Italian observers with the RSVP paradigm. It is well known that, by limiting the role of eye movements, RSVP is particularly effective in capturing the maximum limit of reading performance (Rubin and Turano 1992). In this way, it may be particularly suited to detect individual differences in performance even though other approaches may also provide complementary evidence on this (Marinelli et al. 2014). Again, while quite effective in some respects, the RSVP may not be ideal to detect other components of cross-linguistic differences in reading, including the aim of the present study, i.e., the criterion. Overall, it appears that different approaches and paradigms are most effective in capturing different aspects contributing to the cross-linguistic differences in reading which are present between readers of opaque and transparent orthographies. Indeed, it is unlikely that a single paradigm will be capable of capturing all such differences.

Capitalizing on the pattern of findings coming from such a variety of paradigms, one can try to provide an initial proposal to comprehensively describe the cross-linguistic differences in reading among readers of different orthographies. Readers of transparent orthographies, such as Dutch, German, Italian, or Welsh, learn in a relatively easy fashion the initial rudiments of reading, particularly the correspondences between graphemes and phonemes. In early grades, reading is slow but early on it becomes quite accurate; a tendency to rely on small grain sizes is evident and is marked by a strong length effect which slowly reduces with age (Zoccolotti et al. 2005). Still, even in adults, some role of word length is detected and is characteristically different in readers of German versus English (Ziegler et al. 2001). Good mastering of grapheme–phoneme correspondences leads to an early skill in reading pseudowords. Indeed, it was reported that German children were appreciably better than English children in reading non-words (but not number words or numbers); this difference was particularly marked in the early stages of reading (Wimmer and Goswami 1994). However, there are also several indications that readers of transparent orthographies develop lexical processing, though this occurs at a later ontogenetic stage than the early focus on the acquisition of the non-lexical routine (for a discussion see Paizi et al. 2010).

Readers of an opaque orthography, and in particular English, face an initial, great difficulty in reading (Seymour et al. 2003), presumably because of the lack of bi-univocity of grapheme-to-phoneme mapping and the large portion of words, which present various degrees and forms of irregularity, including irregular words (such as “come” or “pint”) and non-homographic homophones (such as “cite”, “site”, and “sight”). Particularly complex is the identification of diphthongs such as “ea” or “ou” which may be read in several different ways (e.g., compare “ea” in words as “beast”/’bi:st/, “clear” (/’klɪə/), “head”/’hed/, “swear”/’sweə/, early/’ɜ:lɪ/, great/’greɪt/, and heart/’hɑ:t/). Note, in turn, that each of the seven different translations of “ea” (such as /i:/) can have different orthographic solutions (such as “ee” in “heel” or “ei” as in “ceiling”, apart from “ea” as in “beast”). In the presence of such complexity, children may adopt a “lexical” approach, trying to remember and recognize individual words as wholes, or with reference to significant sub-word units such as rhymes or morphemes. Learning grapheme-to-phoneme conversion rules may occur in parallel (or perhaps as a by-product of) with this process. Evidence from different sources is in keeping with this idea. Young English children are better than the Italian peers in learning a lexical pseudoword–figure association task (Marinelli et al. 2020). Reliance on relatively large reading units (such as body rhymes) is noted in various investigations including studies that compare English and German observers (Ziegler et al 2001). Extreme examples of lexically oriented reading come from individual cases of English speakers where word reading is appropriate while reading of pseudowords is highly impaired despite regular school attendance (Howard 1996; Stothard et al. 1996). In these cases, reading in standard clinical tests is within normal limits indicating that, in principle, reasonable reading skills may be achieved even in the absence of a clear acquisition of grapheme-to-phoneme conversion rules. Interestingly, the drive to use a lexically oriented approach may prove difficult to at least a proportion of children, thus generating large individual differences in reading. It has been noted that about one-fourth of English readers are highly deviant in their reading capacity (Spencer and Hanley 2003; Hanley et al. 2004; Marinelli et al. 2016). This may indicate that a sizeable proportion of English children find it difficult to adopt a lexical strategy early on, generating the large individual differences in overall performance which, as indicated above, are detected most clearly by the RVSP (Marinelli et al. 2014).

Overall, we show that in a lexical decision task, English-speaking young adults set their standards for accepting the stimulus at a lower level than Italian observers and show a lower sensitivity selectively for word stimuli. The criterion difference was specific to an orthographic task and did not extend to a face gender discrimination task. We propose that this cross-linguistic difference may stem from the emphasis that the English orthography places from the very early stages of learning on adopting a “lexical” strategy of reading. In keeping with the MROM (Grainger and Jacobs 1996; Grainger 2018), we propose the working hypothesis that this is associated with the adoption of a criterion based on general lexical evidence rather than a criterion based on evidence for a specific word orthographic representation (hypothesized as the principal base for the judgments of Italian observers). The use of a lexical decision task and Ratcliff’s Diffusion model was instrumental in detecting such differences, which may be more difficult to identify in other tasks, including the classical reading of individual words (measuring vocal RTs). Indeed, here we show that a lexical decision task has been instrumental to uncovering the components of the cross-linguistic differences that emerge between transparent versus opaque orthographies.

## Supplementary Information

Below is the link to the electronic supplementary material.

Supplementary file1 (DOCX 71 KB)

## Data Availability

All data generated or analyzed during this study are included in this article.
