# Supplementary material for: Decision times in orthographic processing: a cross-linguistic study
Source: Exp Brain Res. 2023 Jan 11;241(2):585–99. doi: 10.1007/s00221-022-06542-0 (PMC9894970; doi:10.1007/s00221-022-06542-0)
Supplement: Supplementary file 1 — Supplementary file1 (DOCX 71 KB) [file 221_2022_6542_MOESM1_ESM.docx]

**Appendix 1**

The appendix reports the formulas of Mixed effect models.

**Experiment 1**

Linear mixed effect model on raw RTs to correct responses with lexicality (words, pseudowords) and language group (English, Italian) as fixed effects and stimuli and participants as random effects.


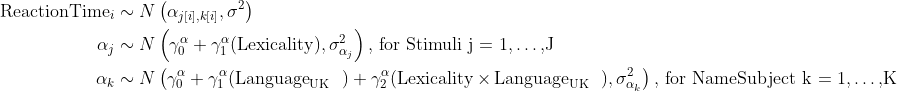


Linear mixed effect model on raw RTs to correct responses with lexicality (words, pseudowords) and language group (English, Italian) as fixed effects, N-size as a covariate, and stimuli and participants as random effects.


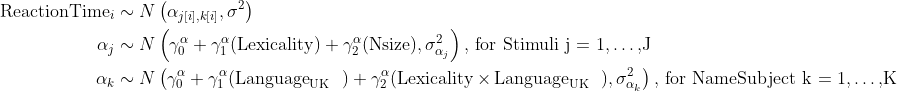


Logistic mixed effect model on reading errors, with lexicality (words, pseudowords) and language group (English, Italian) as fixed effects and stimuli and participants as random effects.


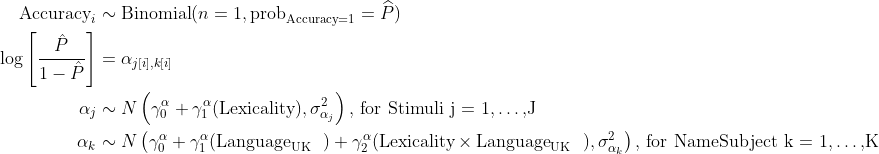


Logistic mixed effect model on reading errors, with lexicality (words, pseudowords) and language group (English, Italian) as fixed effects, N-size as covariate, and stimuli and participants as random effects.


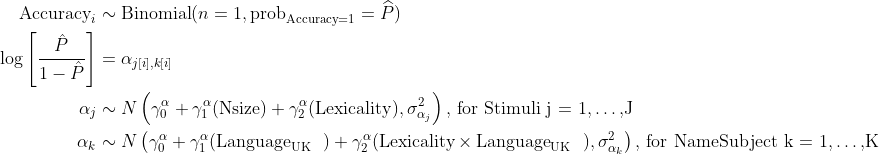


Linear mixed effect model on raw RTs on correct responses with frequency (medium, low) and language group (English, Italian) as fixed effects and stimuli and participants as random effects.


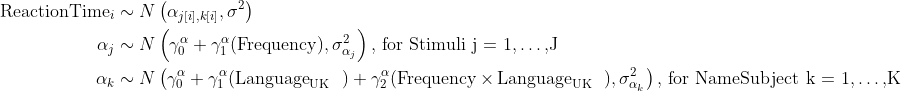


Linear mixed effect model on raw RTs on correct responses with frequency (medium, low) and language group (English, Italian) as fixed effects, N-size as covariate, and stimuli and participants as random effects.


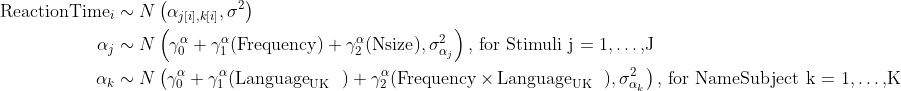


Logistic mixed effect model was performed on accuracy score in reading words, with frequency (medium, low) and language group (English, Italian) as fixed effects and stimuli and participants as random effects.


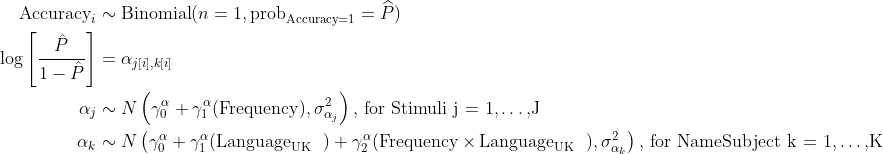


Logistic mixed effect model was performed on accuracy score in reading words, with frequency (medium, low) and language group (English, Italian) as fixed effects, N-size as covariate, and stimuli and participants as random effects.


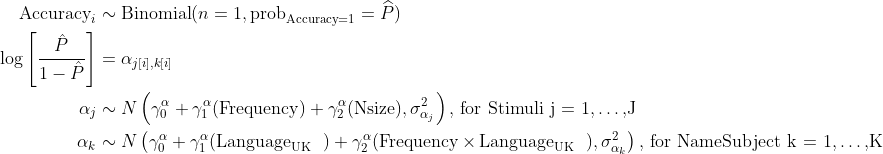


**Experiment 2**

Linear mixed effect model on raw RTs to correct responses with language (English, Italian), face gender (male, female), and order of stimulus repetition as fixed factors, and items and participants as random factors.

$${ReactionTime}_{i}\sim N(\mu,\sigma2)$$

$$\mu=\alpha j\left[ i \right],k\left[ i \right]+\beta1\left( OrderRCB \right)+\beta2\left( OrderRCC \right)+\beta3\left( OrderRCD \right)+\beta4\left( OrderRCE \right)+\beta5\left( OrderRCF \right)+\beta6\left( OrderRCG \right)+\beta7\left( OrderRCH \right)+\beta8\left( OrderRCI \right)+\beta9\left( OrderRCJ \right)+\beta10\left( OrderRCK \right)+\beta11\left( OrderRCL \right)+\beta12\left( OrderRCM \right)+\beta13\left( OrderRCN \right)+\beta14\left( OrderRCO \right)+\beta15\left( OrderRCP \right)+\beta16\left( OrderRCQ \right)+\beta17\left( OrderRCR \right)+\beta18\left( OrderRCS \right)+\beta19\left( OrderRCT \right)+\beta20\left( OrderRCU \right)+\beta21\left( OrderRCV \right)+\beta22\left( OrderRCW \right)$$

$$\alpha j \sim N(\gamma0\alpha+\gamma1\alpha(LanguageITA)+\gamma2\alpha(LanguageITA\times OrderRCB)+\gamma3\alpha(LanguageITA\times OrderRCC)+\gamma4\alpha(LanguageITA\times OrderRCD)+\gamma5\alpha(LanguageITA\times OrderRCE)+\gamma6\alpha(LanguageITA\times OrderRCF)+\gamma7\alpha(LanguageITA\times OrderRCG)+\gamma8\alpha(LanguageITA\times OrderRCH)+\gamma9\alpha(LanguageITA\times OrderRCI)+\gamma10\alpha(LanguageITA\times OrderRCJ)+\gamma110\alpha(LanguageITA\times OrderRCK)+\gamma121\alpha(LanguageITA\times OrderRCL)+\gamma13\alpha(LanguageITA\times OrderRCM)+\gamma14\alpha(LanguageITA\times OrderRCN)+\gamma15\alpha(LanguageITA\times OrderRCO)+\gamma16\alpha(LanguageITA\times OrderRCP)+\gamma17\alpha(LanguageITA\times OrderRCQ)+\gamma18\alpha(LanguageITA\times OrderRCR)+\gamma19\alpha(LanguageITA\times OrderRCS)+\gamma20\alpha(LanguageITA\times OrderRCT)+\gamma21\alpha(LanguageITA\times OrderRCU)+\gamma22\alpha(LanguageITA\times OrderRCV)+\gamma23\alpha(LanguageITA\times OrderRCW),\sigma\alpha j2), for SubName j = 1,\ldots,J$$

$$\alpha k \sim N(\gamma0\alpha+\gamma1\alpha(Gendermale )+\gamma2\alpha(Gendermale \times OrderRCB)+\gamma3\alpha(Gendermale \times OrderRCC)+\gamma4\alpha(Gendermale \times OrderRCD)+\gamma5\alpha(Gendermale \times OrderRCE)+\gamma6\alpha(Gendermale \times OrderRCF)+\gamma7\alpha(Gendermale \times OrderRCG)+\gamma8\alpha(Gendermale \times OrderRCH)+\gamma9\alpha(Gendermale \times OrderRCI)+\gamma10\alpha(Gendermale \times OrderRCJ)+\gamma110\alpha(Gendermale \times OrderRCK)+\gamma121\alpha(Gendermale \times OrderRCL)+\gamma132\alpha(Gendermale \times OrderRCM)+\gamma143\alpha(Gendermale \times OrderRCN)+\gamma154\alpha(Gendermale \times OrderRCO)+\gamma165\alpha(Gendermale \times OrderRCP)+\gamma176\alpha(Gendermale \times OrderRCQ)+\gamma187\alpha(Gendermale \times OrderRCR)+\gamma198\alpha(Gendermale \times OrderRCS)+\gamma209\alpha(Gendermale \times OrderRCT)+\gamma210\alpha(Gendermale \times OrderRCU)+\gamma221\alpha(Gendermale \times OrderRCV)+\gamma232\alpha(Gendermale \times OrderRCW)+\gamma24\alpha(Gendermale \times LanguageITA)+\gamma25\alpha(Gendermale \times LanguageITA\times OrderRCB)+\gamma26\alpha(Gendermale \times LanguageITA\times OrderRCC)+\gamma27\alpha(Gendermale \times LanguageITA\times OrderRCD)+\gamma28\alpha(Gendermale \times LanguageITA\times OrderRCE)+\gamma29\alpha(Gendermale \times LanguageITA\times OrderRCF)+\gamma30\alpha(Gendermale \times LanguageITA\times OrderRCG)+\gamma31\alpha(Gendermale \times LanguageITA\times OrderRCH)+\gamma32\alpha(Gendermale \times LanguageITA\times OrderRCI)+\gamma33\alpha(Gendermale \times LanguageITA\times OrderRCJ)+\gamma34\alpha(Gendermale \times LanguageITA\times OrderRCK)+\gamma35\alpha(Gendermale \times LanguageITA\times OrderRCL)+\gamma36\alpha(Gendermale \times LanguageITA\times OrderRCM)+\gamma37\alpha(Gendermale \times LanguageITA\times OrderRCN)+\gamma38\alpha(Gendermale \times LanguageITA\times OrderRCO)+\gamma39\alpha(Gendermale \times LanguageITA\times OrderRCP)+\gamma40\alpha(Gendermale \times LanguageITA\times OrderRCQ)+\gamma41\alpha(Gendermale \times LanguageITA\times OrderRCR)+\gamma42\alpha(Gendermale \times LanguageITA\times OrderRCS)+\gamma43\alpha(Gendermale \times LanguageITA\times OrderRCT)+\gamma44\alpha(Gendermale \times LanguageITA\times OrderRCU)+\gamma45\alpha(Gendermale \times LanguageITA\times OrderRCV)+\gamma46\alpha(Gendermale \times LanguageITA\times OrderRCW),\sigma\alpha k2), for imagename k = 1,\ldots,K$$

Logistic mixed effect model on accuracy score with language (English, Italian), face gender (male, female), and order of stimulus repetition as fixed factors, and items and participants as random factors.

Accuracy*i*​ ​​ ∼Binomial(*n*=1,probAccuracy=1​=*P*)

$$\log\left[ \frac{\hat{P}}{1-\hat{P}} \right] =\alpha j[i],k[i]+\beta1(\mathrm{OrderRCB})+\beta2(\mathrm{OrderRCC})+\beta3(\mathrm{OrderRCD})+\beta4(\mathrm{OrderRCE})+\beta5(\mathrm{OrderRCF})+\beta6(\mathrm{OrderRCG})+\beta7(\mathrm{OrderRCH})+\beta8(\mathrm{OrderRCI})+\beta9(\mathrm{OrderRCJ})+\beta10(\mathrm{OrderRCK})+\beta11(\mathrm{OrderRCL})+\beta12(\mathrm{OrderRCM})+\beta13(\mathrm{OrderRCN})+\beta14(\mathrm{OrderRCO})+\beta15(\mathrm{OrderRCP})+\beta16(\mathrm{OrderRCQ})+\beta17(\mathrm{OrderRCR})+\beta18(\mathrm{OrderRCS})+\beta19(\mathrm{OrderRCT})+\beta20(\mathrm{OrderRCU})+\beta21(\mathrm{OrderRCV})+\beta22(\mathrm{OrderRCW})$$

$$\alpha j \sim N(\gamma0\alpha+\gamma1\alpha(LanguageITA)+\gamma2\alpha(LanguageITA\times OrderRCB)+\gamma3\alpha(LanguageITA\times OrderRCC)+\gamma4\alpha(LanguageITA\times OrderRCD)+\gamma5\alpha(LanguageITA\times OrderRCE)+\gamma6\alpha(LanguageITA\times OrderRCF)+\gamma7\alpha(LanguageITA\times OrderRCG)+\gamma8\alpha(LanguageITA\times OrderRCH)+\gamma9\alpha(LanguageITA\times OrderRCI)+\gamma10\alpha(LanguageITA\times OrderRCJ)+\gamma110\alpha(LanguageITA\times OrderRCK)+\gamma121\alpha(LanguageITA\times OrderRCL)+\gamma13\alpha(LanguageITA\times OrderRCM)+\gamma14\alpha(LanguageITA\times OrderRCN)+\gamma15\alpha(LanguageITA\times OrderRCO)+\gamma16\alpha(LanguageITA\times OrderRCP)+\gamma17\alpha(LanguageITA\times OrderRCQ)+\gamma18\alpha(LanguageITA\times OrderRCR)+\gamma19\alpha(LanguageITA\times OrderRCS)+\gamma20\alpha(LanguageITA\times OrderRCT)+\gamma21\alpha(LanguageITA\times OrderRCU)+\gamma22\alpha(LanguageITA\times OrderRCV)+\gamma23\alpha(LanguageITA\times OrderRCW),\sigma\alpha j2), for SubName j = 1,\ldots,J$$

$$\alpha k\sim N(\gamma0\alpha+\gamma1\alpha(GenderMale )+\gamma2\alpha(GenderMale \times OrderRCB)+\gamma3\alpha(GenderMale \times OrderRCC)+\gamma4\alpha(GenderMale \times OrderRCD)+\gamma5\alpha(GenderMale \times OrderRCE)+\gamma6\alpha(GenderMale \times OrderRCF)+\gamma7\alpha(GenderMale \times OrderRCG)+\gamma8\alpha(GenderMale \times OrderRCH)+\gamma9\alpha(GenderMale \times OrderRCI)+\gamma10\alpha(GenderMale \times OrderRCJ)+\gamma110\alpha(GenderMale \times OrderRCK)+\gamma121\alpha(GenderMale \times OrderRCL)+\gamma132\alpha(GenderMale \times OrderRCM)+\gamma143\alpha(GenderMale \times OrderRCN)+\gamma154\alpha(GenderMale \times OrderRCO)+\gamma165\alpha(GenderMale \times OrderRCP)+\gamma176\alpha(GenderMale \times OrderRCQ)+\gamma187\alpha(GenderMale \times OrderRCR)+\gamma198\alpha(GenderMale \times OrderRCS)+\gamma209\alpha(GenderMale \times OrderRCT)+\gamma210\alpha(GenderMale \times OrderRCU)+\gamma221\alpha(GenderMale \times OrderRCV)+\gamma232\alpha(GenderMale \times OrderRCW)+\gamma24\alpha(GenderMale \times LanguageITA)+\gamma25\alpha(GenderMale \times LanguageITA\times OrderRCB)+\gamma26\alpha(GenderMale \times LanguageITA\times OrderRCC)+\gamma27\alpha(GenderMale \times LanguageITA\times OrderRCD)+\gamma28\alpha(GenderMale \times LanguageITA\times OrderRCE)+\gamma29\alpha(GenderMale \times LanguageITA\times OrderRCF)+\gamma30\alpha(GenderMale \times LanguageITA\times OrderRCG)+\gamma31\alpha(GenderMale \times LanguageITA\times OrderRCH)+\gamma32\alpha(GenderMale \times LanguageITA\times OrderRCI)+\gamma33\alpha(GenderMale \times LanguageITA\times OrderRCJ)+\gamma34\alpha(GenderMale \times LanguageITA\times OrderRCK)+\gamma35\alpha(GenderMale \times LanguageITA\times OrderRCL)+\gamma36\alpha(GenderMale \times LanguageITA\times OrderRCM)+\gamma37\alpha(GenderMale \times LanguageITA\times OrderRCN)+\gamma38\alpha(GenderMale \times LanguageITA\times OrderRCO)+\gamma39\alpha(GenderMale \times LanguageITA\times OrderRCP)+\gamma40\alpha(GenderMale \times LanguageITA\times OrderRCQ)+\gamma41\alpha(GenderMale \times LanguageITA\times OrderRCR)+\gamma42\alpha(GenderMale \times LanguageITA\times OrderRCS)+\gamma43\alpha(GenderMale \times LanguageITA\times OrderRCT)+\gamma44\alpha(GenderMale \times LanguageITA\times OrderRCU)+\gamma45\alpha(GenderMale \times LanguageITA\times OrderRCV)+\gamma46\alpha(GenderMale \times LanguageITA\times OrderRCW),\sigma\alpha k2), for imagename k = 1,\ldots,K$$
